# Strong association between genomic 3D structure and CRISPR cleavage efficiency

**DOI:** 10.1371/journal.pcbi.1012214

**Published:** 2024-06-07

**Authors:** Shaked Bergman, Tamir Tuller

**Affiliations:** 1 Department of Biomedical Engineering, Tel-Aviv University, Tel Aviv, Israel; 2 The Sagol School of Neuroscience, Tel-Aviv University, Tel Aviv, Israel; Children’s National Hospital, George Washington University, UNITED STATES

## Abstract

CRISPR is a gene editing technology which enables precise in-vivo genome editing; but its potential is hampered by its relatively low specificity and sensitivity. Improving CRISPR’s on-target and off-target effects requires a better understanding of its mechanism and determinants. Here we demonstrate, for the first time, the chromosomal 3D spatial structure’s association with CRISPR’s cleavage efficiency, and its predictive capabilities. We used high-resolution Hi-C data to estimate the 3D distance between different regions in the human genome and utilized these spatial properties to generate 3D-based features, characterizing each region’s density. We evaluated these features based on empirical, in-vivo CRISPR efficiency data and compared them to 425 features used in state-of-the-art models. The 3D features ranked in the top 13% of the features, and significantly improved the predictive power of LASSO and xgboost models trained with these features. The features indicated that sites with lower spatial density demonstrated higher efficiency. Understanding how CRISPR is affected by the 3D DNA structure provides insight into CRISPR’s mechanism in general and improves our ability to correctly predict CRISPR’s cleavage as well as design sgRNAs for therapeutic and scientific use.

## Author summary

CRISPR is an efficient and precise genome editing technology; but our understanding of the factors influencing it, and our ability to accurately design and predict its efficiency at different target sites, are lacking. Current models mostly use the sequence of the target site, rather than incorporating additional information regarding its genomic location. Here we propose a new type of predictive feature, based on the 3D structure of the genome. We show that features estimating the spatial density of the target site correlate highly with CRISPR efficiency, compared to classic sequence-based features; and that adding these features to predictive models significantly improves their power. CRISPR efficiency was negatively correlated with 3D density, indicating CRISPR is more efficient in sparse regions–possibly due to easier access to the target site. Improving our ability to predict CRISPR action will allow us to further understand its mechanism and better utilize it in research and medicine.

## Introduction

Genome-editing technologies are on the rise. Chief among them is CRISPR, which is used to induce DNA mutations in precise genomic locations (reviewed in [1]). CRISPR’s precision and ease-of-use, relative to other genome editing technologies, make it a valuable therapeutic and scientific tool: it provides a powerful method to conduct basic research as well as develop treatments for diseases such as cancer, AIDS and many others (reviewed in [2,3]).

The CRISPR complex consists of a short Single Guide RNA (sgRNA) and an endonuclease (e.g. Cas9). The sgRNA binds to a complementary 20nt target site in the DNA, and upon this binding, the nuclease induces a double-stranded break (DSB) in the DNA. The cell repairs the break–and in many cases does so imperfectly, introducing mutations to the cut site in the process. Thus, a precise DSB combined with a prediction of its resultant mutation could serve as a way to edit genomes.

An ideal sgRNA would have both high sensitivity (i.e. will successfully affect the intended target site) and high specificity (i.e. will not affect any unintended sites). However, despite significant improvements to our knowledge regarding CRISPR’s mechanism and influencing factors, the design of such a sgRNA remains a challenge. Several computational tools to predict CRISPR efficiency [4–24], predict CRISPR’s repair outcomes [25–29], and aid in the design of gRNAs [30–49] were developed over the last decade, but their sensitivity and specificity are relatively low (reviewed in [50–55]). This may be partly due to the mainly local features these models use: the GC content and the nucleotide identity of target site position, as well as thermodynamic features such as the melting temperature of the DNA target site and the free energy of the sgRNA. Only a few models utilize epigenetic features [5,10,21,23], and further complementing this picture with global, cell-dependent features would provide better understanding of the determinants of CRISPR’s action. This, in turn, will allow us to design more precise and effective sgRNAs.

DNA is folded in 3D space. Genomic locations which are far away from each other in 1D (i.e. when considering only their chromosomal coordinates) may be close to one another in 3D, inducing common characteristics which cannot be found when considering only the 1D genome. Indeed, the spatial configuration of the DNA was shown to influence several cellular processes, such as differentiation and gene expression (reviewed in [56–59]); nevertheless, this aspect was not studied before in relation to CRISPR cleavage.

**Fig 1:**
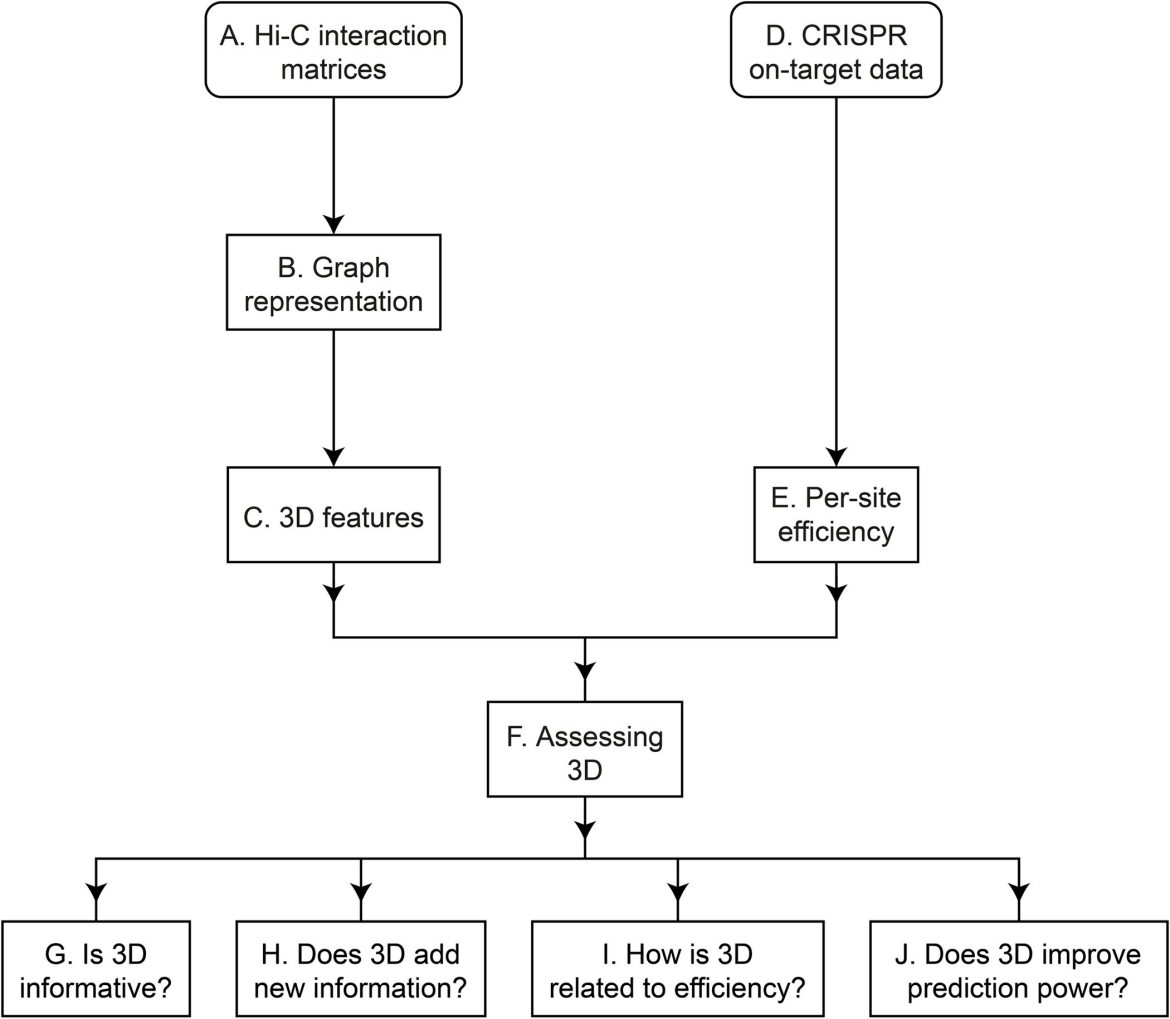
The general structure of the study. See details in the main text.

Here we highlight, for the first time, the effect of the genome’s 3D structure on CRISPR efficiency (Fig 1). We show that the cleavage efficiency in spatially dense regions tends to be lower than in sparse regions, and that using the 3D DNA density significantly improves prediction of CRISPR efficiency.

## Results

The study is summarized in Fig 1. We acquired Hi-C interaction matrices and CRISPR efficiency data, to explore the relation between the two. We refer here to the boxes in Fig 1, and further detail each step in the next sections.

Based on the Hi-C data (Fig 1A), we represented each human chromosome as a weighted graph (Fig 1B), where each node is a genomic region, and each edge is the distance between two regions. Based on these graphs, we calculated local 3D features for each genomic coordinate (Fig 1C).

Then, using the CRISPR data (Fig 1D), we estimated the efficiency for each site (Fig 1E), calculated the features for all the target sites, and assessed the relation between our 3D features and CRISPR efficiency in the following ways (Fig 1F): we compared our features to classic, well-established features that are used in the vast majority of CRISPR prediction models (Fig 1G); we checked whether our features add new information relative to state-of-the-art computational models (Fig 1H); checked whether there is a consistent type of relation between the local 3D structure and CRISPR efficiency, and what is its nature (Fig 1I); and finally, we showed that adding our features significantly improved the prediction power of a model trained on the established CRISPR features (Fig 1J).

### From Hi-C data to 3D-distance features

We set out to generate a 3D structure of the human genome, by dividing each chromosome to 10kb regions (“bins”) and estimating the distance between each pair of bins. To that end, we downloaded per-chromosome contact matrices for T cells [60] and HEK293 [61]; these matrices were created from high-resolution Hi-C (10kb) and denote the number of interactions between each two bins; we used the normalized number of interactions as our data.

Briefly, we first represented each chromosomal interaction matrix as a mathematical graph; then we generated, for each chromosome, a distance matrix based on the p shortest paths between each pair of bins (p = 5). The usage of graph paths was intended to smooth the distance patterns: we did not simply take the interaction frequency between two nodes as their distance, but rather took into consideration the graph paths between them and defined the average length of the 5 shortest paths with up to 2 edges as the distance between them. Thus, we used up to 10 normalized interaction frequencies when estimating the distance between a single pair of nodes.

This way, we procured 3D-distance matrices; the next step was calculating 3D-based features, to capture the spatial properties of each genomic bin. For each genomic bin B, we generated two sets of features: “Distance (n)” and “Radius (m)”. For the “Distance (n)” features, we calculated the average distance between bin B and the n closest bins in the chromosome; for the “Radius (m)” features, we counted the number of bins whose distance to B is shorter than a certain radius (see “Calculating 3D-based features”). Both sets measure an aspect of spatial density, complementing one another in its assessment: “Distance (n)” is expected to be inversely proportional to spatial density, since a long distance between B and the bins closest to it indicates that the immediate vicinity of bin B is empty; on the other hand, “Radius (m)” is positively proportional to density, as a higher number of bins within a fixed radius from bin B indicates a higher density. In the main body of this work, we focus on the performance of a single representative feature for each CRISPR dataset: “Distance (2500)” for the Leenay dataset, “Distance (1000)” for the TTISS dataset and “Radius (10)” for the GUIDE-Seq dataset. The values of these features, for all human genomic bins, are available in S1 Table.

### Acquiring in-vivo CRISPR efficiency dataset

To evaluate our 3D-based features we required in-vivo CRISPR efficiency data, which would both be measured in-vivo in and include information regarding the target site’s location. Most large CRISPR datasets are either measured in-vitro, or don’t detail the genomic location of the target sites (e.g. [8,62]); the largest such datasets are the Leenay, TTISS and GUIDE-Seq datasets [27,63,64]. These are the CRISPR datasets we use in the following sections. The Leenay dataset includes on-target efficiency data for 1574 genomic locations in T cells, and the TTISS dataset includes off-target efficiency data for 666 genomic locations in HEK293 cells. The GUIDE-Seq dataset includes 260 U2OS off-targets and 153 HEK293 sites. Since we could not find U2OS Hi-C data with sufficient depth, we used the GUIDE-Seq HEK293 dataset in our main analyses, and the U2OS in a cross-cell analysis (see “3D features could be used to improve predictive models in non-matching cell types”).

### Feature signal is reduced when considering lower resolutions

Before comparing our features to the state-of-the-art features and models, we wanted to isolate the effect of the Hi-C resolution on their performance; in addition to the 10kb resolution described in the previous sections, we generated features based on two other common resolutions: 25kb and 50kb (S1 Fig). We also found, for each site, whether it resides in a TAD (in 100kb resolution), the corresponding TAD’s length, and whether it is in an A or B compartment (in 1Mb resolution). The correlations between our features and CRISPR efficiency were substantially reduced in the Leenay and TTISS datasets. In the GUIDE-Seq dataset, slightly higher maximal correlations were achieved using 25kb and 50kb resolutions (0.14 and 0.12, respectively, vs. 0.11 in the 10kb resolution), but the average correlation remained virtually the same (0.08 for all three resolutions). The TAD and A/B compartment features performed worse than the 10kb features in all 3 datasets. Overall, the 10kb resolution was the most robust over the 3 datasets, underlining the need for high-resolution data for our features. Thus, the rest of our analysis concerns the features based on 10kb Hi-C data.

### 3D features perform comparably to classic predictive features

**Fig 2:**
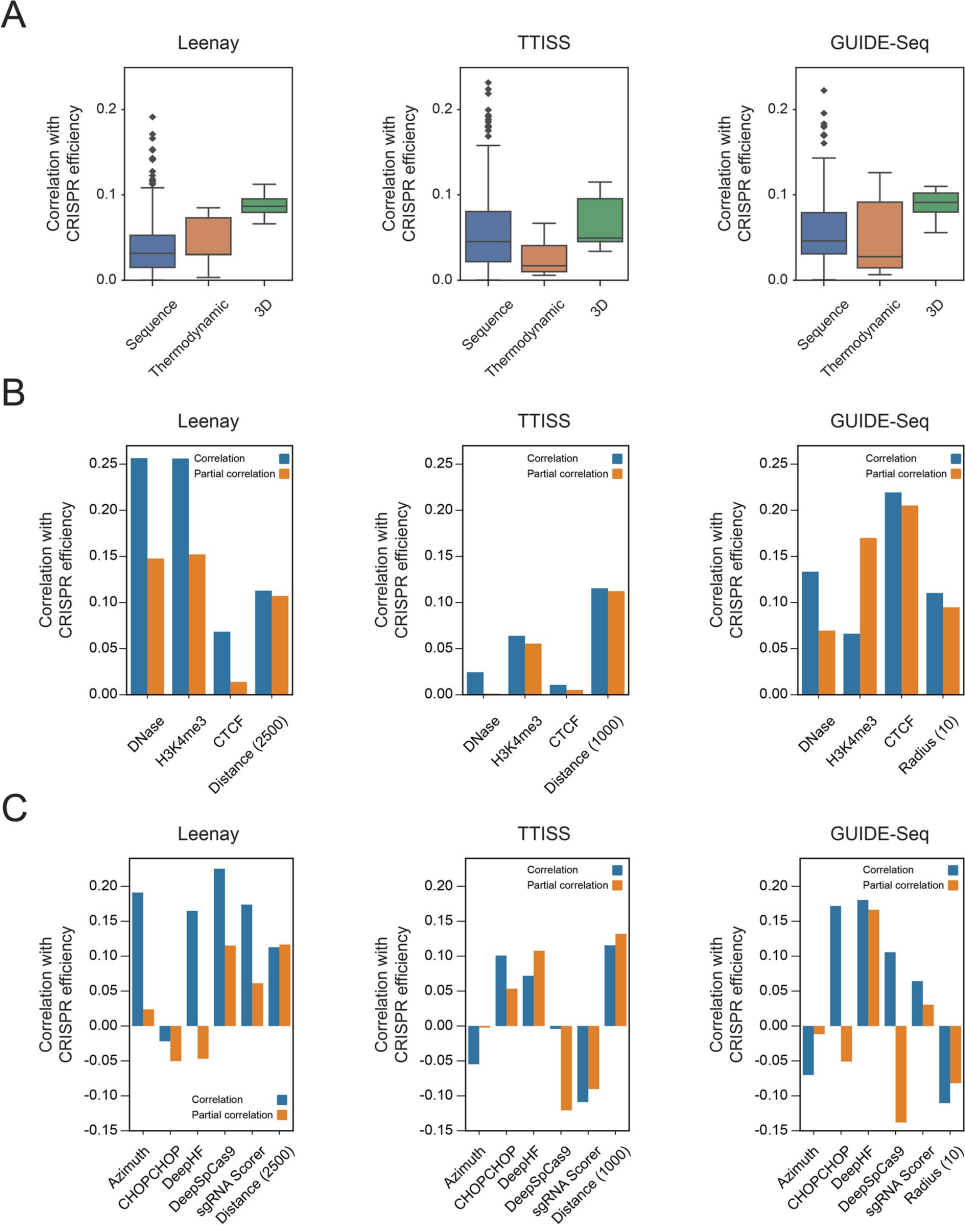
3D features perform comparably to state-of-the-art features and encode new information. (A) Boxplots of Pearson correlations (absolute values) between predictive features and CRISPR efficiency in the Leenay, TTISS and GUIDE-Seq datasets. Blue: 420 sequence features; orange: 5 thermodynamic features; green: 9 3D features. (B-C) Bar charts of Pearson correlations (blue) and partial correlations (orange) with CRISPR efficiency, in the Leenay, TTISS and GUIDE-Seq datasets. The partial correlation of each vector controls for all other vectors in the dataset’s subplot. (B) Epigenetic features and 3D feature (correlations appear in absolute value); (C) State-of-the-art models and 3D feature.

To assess whether our features are informative, we compared them to “classic” predictors of CRISPR efficiency. The main category of features used in CRISPR models (e.g. [4,8,17,20,62,65]) is sequence features: the identity of the nucleotide/dinucleotide at each position along the target site, as well as the distribution of nucleotides/dinucleotides in the site as a whole. Another established category of features is thermodynamics: the melting temperature of the DNA target site and the free energy of the sgRNA. We calculated a total of 420 sequence features and 5 thermodynamic features on the CRISPR efficiency datasets (S2 Table) and compared these 425 features to our nine 3D features (Fig 2A). In the Leenay dataset, four of the “Distance” features (using 4 different cutoffs) were ranked in the top 30 correlations out of the 434, with the highest, “Distance (2500)”, being ranked in the 18th place (top 5%). Notably, both our “Distance” (17th, r = 0.11, see S2 Fig) and “Radius” (35th, r = 0.08) features outperformed the thermodynamic features, whose top feature was ranked 38th (r = 0.08). In the TTISS dataset, the top 3D feature was “Distance (1000)”, which was ranked in the 53rd place (top 13%, r = 0.11, see S2 Fig); the 3D features outperformed the thermodynamic features here as well. In the GUIDE-Seq dataset, the top 3D feature was “Radius (10)”, ranked 34th (top 8%, r = 0.11; see S2 Fig). While the best thermodynamic feature had a higher correlation (0.13), the average 3D feature correlation was higher than the average thermodynamic correlation (0.09 vs 0.05). The average correlation of the classic feature set was 0.04/0.06/0.06 in the Leenay/TTISS/GUIDE-Seq dataset, respectively. Thus, the 3D features are potentially valuable features when compared to the features which are widely used today. For simplicity’s sake, in the next sections we will evaluate the information contained in the best representative feature of the set we have generated–“Distance (2500)”, in the Leenay dataset, “Distance (1000)” in the TTISS dataset and “Radius (10)” in the GUIDE-Seq dataset. We note that this inconsistency in the ranking of correlations was observed in the sequence and thermodynamic features as well: only one sequence feature was ranked in the top 10 features in more than one dataset; and the thermodynamic feature ranking differed for each dataset (no single feature was ranked in the first two places for all three datasets).

### The correlation between 3D structure and CRISPR efficiency is not explained by gene expression

Gene expression was previously shown to be highly associated with the 3D structure of the genome [66,67]. Thus, we investigated whether it could at least partially explain the 3D-CRISPR correlation we observed; we downloaded expression levels for T cells and HEK293 from the Expression Atlas website [68].

For each dataset, we determined which CRISPR target sites resided in coding genes; 653 / 359 / 95 such sites were found out of 1574 / 666 / 153 sites in the Leenay / TTISS / GUIDE-Seq dataset, respectively. For these sites, we calculated the Spearman correlation and partial correlation of the representative 3D feature with the CRISPR efficiency, controlling for the expression levels of the genes (in the corresponding cell type). The full Spearman correlation between the 3D feature and CRISPR efficiency was 0.079 / 0.146 / -0.1, while the partial correlation was almost identical: 0.08 / 0.149 / -0.1 for the Leenay / TTISS / GUIDE-Seq dataset, respectively. Thus, since controlling for gene expression does not diminish the relation between 3D density and CRISPR efficiency, this relation cannot be attributed to gene expression as a confounding variable.

### 3D feature adds new information not encoded in epigenetic features and state-of-the-art efficiency models

After observing that our features are indeed informative and correlative with CRISPR efficiency, we checked whether they provide new information that is not contained in current epigenetic features and state-of-the-art models.

We calculated 3 epigenetic features, assessing the chromatin accessibility of different positions in the genome based on DNase, H3K4me3 and CTCF (see details in S1 Text and S3 Table). We then calculated the correlation and partial correlation between CRISPR efficiency and 4 features: the 3D feature and 3 epigenetic features (Fig 2B). The partial correlation controlled for the other 3 features assessed (e.g. the partial correlation between DNase and CRISPR efficiency controlled for CTCF, H3K4me3 and the 3D feature). In all three datasets, the 3D feature’s partial correlation was similar to the full correlation (both the full correlation and partial correlations were 0.09–0.11 in all datasets). In the Leenay dataset, the partial correlation for the 3 epigenetic features was lower than the full correlation but remained at around 0.15 for DNase and H3K4me3. In the TTISS dataset, the epigenetic features’ correlations were low to begin with, and remained low–both relative to the full correlation, and relative to the 3D feature’s correlation. In the GUIDE-Seq dataset, the partial correlation for two of the three epigenetic features was lower than the full correlation but remained at around 0.15 for H3K4me3 and CTCF. Thus, controlling for the epigenetic features did not significantly reduce the 3D feature’s correlation, meaning the 3D features encode information not included in the epigenetic features. We can also conclude from these data that the epigenetic features encode some similar information to one another, but since controlling for one does not nullify the other, the epigenetic features could be used in tandem with the 3D feature.

We next compared our representative 3D features to 5 state-of-the-art CRISPR efficiency models: Azimuth [62], DeepSpCas9 [17], CHOPCHOP [40] (using the Moreno-Mateos [4], i.e. CRISPRscan, scoring scheme), DeepHF [8] and sgRNA Scorer [20]. The models are summarized in S4 Table. We calculated the 5 models’ CRISPR efficiency predictions on both CRISPR datasets, thereby acquiring 6 score vectors: 1 vector of 3D feature values and 5 vectors of model predictions. As with the epigenetic features, we calculated each vector’s correlation with CRISPR efficiency, as well as the partial correlation of each vector with CRISPR efficiency when controlling for the other 5 vectors; for example, the partial correlation between DeepSpCas9 and CRISPR efficiency controlled for Azimuth, CHOPCHOP, DeepHF, sgRNA Scorer and the 3D feature (Fig 2C). The partial correlations of the 5 models were, generally, significantly lower relative to their correlations with CRISPR efficiency–indicating they encode similar information to one another; whereas the 3D feature’s partial correlation remained almost the same (full correlation 0.11 / 0.12 / -0.11 vs. partial correlation 0.12 / 0.13 / -0.08 in the Leenay / TTISS / GUIDE-Seq dataset, respectively), indicating it indeed adds new information that cannot be explained by the current models.

### 3D density is inversely proportional to CRISPR efficiency

We examined the nature of the relation between our features and CRISPR efficiency; specifically, we inspected whether sites with higher efficiency tended to have a higher or lower spatial density than low-efficiency sites, and whether this trend is consistent among the different features. By comparing the sites with the top 20% efficiency to the sites with bottom 20% efficiency, we found that their respective densities are significantly different in the Leenay dataset (Wilcoxon rank-sum test), and that the direction of the link is consistent among our features and cutoffs (S3 Fig): sites with high efficiency tended to reside in low-density areas. This is reflected both in the “Distance” features, which were higher (i.e. low density) in sites with high efficiency; and in the “Radius” features, which were higher (i.e. high density) in sites with low efficiency. While the difference in densities was not found as significant in the TTISS dataset, and was found as significant in 3 of the 9 features in the GUIDE-Seq dataset, the relationship remained consistent: the average density of low-efficiency sites was higher than for high-efficiency sites for all 9 features by up to 7% / 28% in the TTISS / GUIDE-Seq dataset, respectively. We also note that none of the thermodynamic features could significantly differentiate between the high-efficiency and low-efficiency sites as well; and that when conducting this test on the TTISS CRISPR dataset using the “Distance 1000 / 2500” features generated from Hi-C in T cell, the difference was indeed significant (p < 0.05). These relationships are also consistent with the directions of the correlations between our features and CRISPR efficiency. Thus, 3D density is negatively correlated with CRISPR activity.

### Adding a 3D feature significantly improves CRISPR predictive power

**Fig 3:**
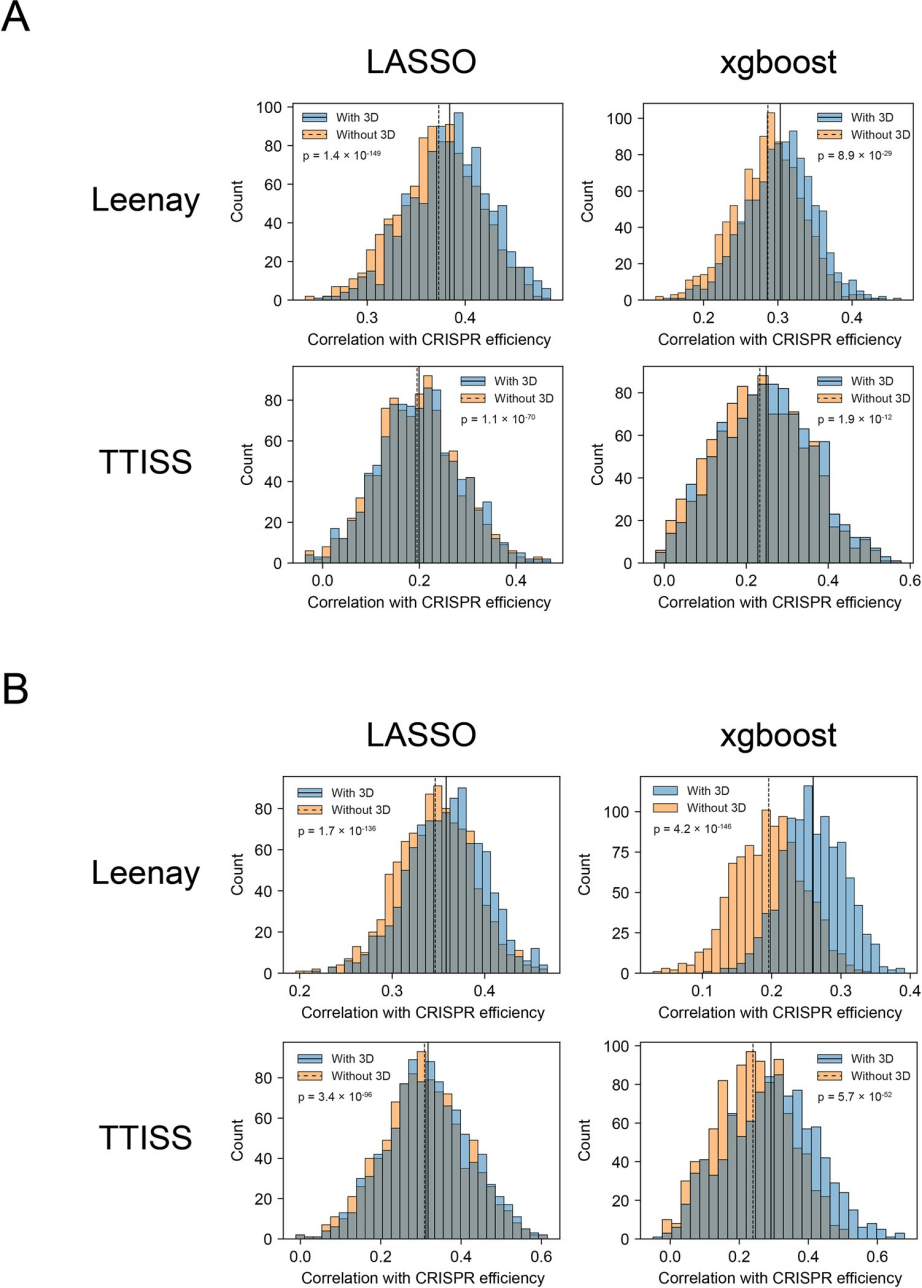
3D features significantly improve CRISPR prediction. Histograms of the correlations between measured and predicted efficiency in the Leenay and TTISS datasets, when testing LASSO and xgboost models. The blue/orange histogram indicates the model with/without the 3D feature and the average correlation is marked with a solid/dashed line, respectively. p-values were calculated using Wilcoxon’s signed rank test. (A) Models using all 425 classic features; (B) Models using the top 30 features, based on Pearson correlation with CRISPR efficiency.

We checked whether adding our 3D feature to a predictive model would improve our ability to predict CRISPR efficiency. To that end, we used the 425 “classic” features, described in the previous sections, to train and assess a predictive model; and then we trained and assessed the same model when adding a single 3D feature. We assessed the models using repeated 5-fold cross-validation, with 200 repeats. We began by conducting this analysis on the Leenay and TTISS datasets, each of which has a sufficient number of observations enough for model assessment (compared to the GUIDE-Seq dataset, which includes 153 observations). In each iteration, we separately trained a model without the 3D feature, and a model with the 3D feature; thus, we acquired two paired distributions of 1000 correlations each. We used two types of predictive models: LASSO regression, and gradient boosting using the popular xgboost algorithm [69]. Adding the 3D feature significantly improved the predictive power of both algorithms (Fig 3A): the average r^2^ of the LASSO model increased by 6% in the Leenay dataset, and 3.9% in the TTISS dataset (p = 1.4 × 10^−149^, and p = 1.1 × 10^−70^, respectively; Two-sided Wilcoxon’s signed rank test between the correlations), and the average r^2^ of the xgboost algorithm increased by 11.8%/11.3% in the Leenay/TTISS dataset, respectively (p = 8.9 × 10^−29^/ p = 1.9 × 10^−12^). The RMSE of the models including a 3D feature was also significantly lower than the models without a 3D feature, for both datasets and model types (S4A Fig).

Due to the large number of features compared to the number of observations, we also conducted this analysis on a reduced feature set, using the top 30 features (based on their Pearson correlation with CRISPR efficiency) instead of the full set of 425 features. Model performance was significantly improved in this case as well–with an r^2^ increase of 6.8% / 5.4% (p = 1.7 × 10^−136^ / p = 3.4 × 10^−96^) in the LASSO model, and a 70.3% / 47.6% increase (p = 4.2 × 10^−146^ / p = 5.7 × 10^−52^) in the xgboost model, for the Leenay / TTISS dataset, respectively (Fig 3B). The RMSE of the models including a 3D feature was significantly lower here as well (S4B Fig).

We then repeated this analysis on the GUIDE-Seq dataset; the trained models were highly overfit, even before using the 3D feature, likely due to the small size of the dataset (n = 153); see additional details in S1 Text and S5 Fig. Thus, we did not continue with the full analysis described in the previous sections for this dataset.

In addition to the improvement of the predictions themselves, we considered the selection and importance of the 3D feature. The 3D feature rankings are summarized in S5 Table.

**Fig 4:**
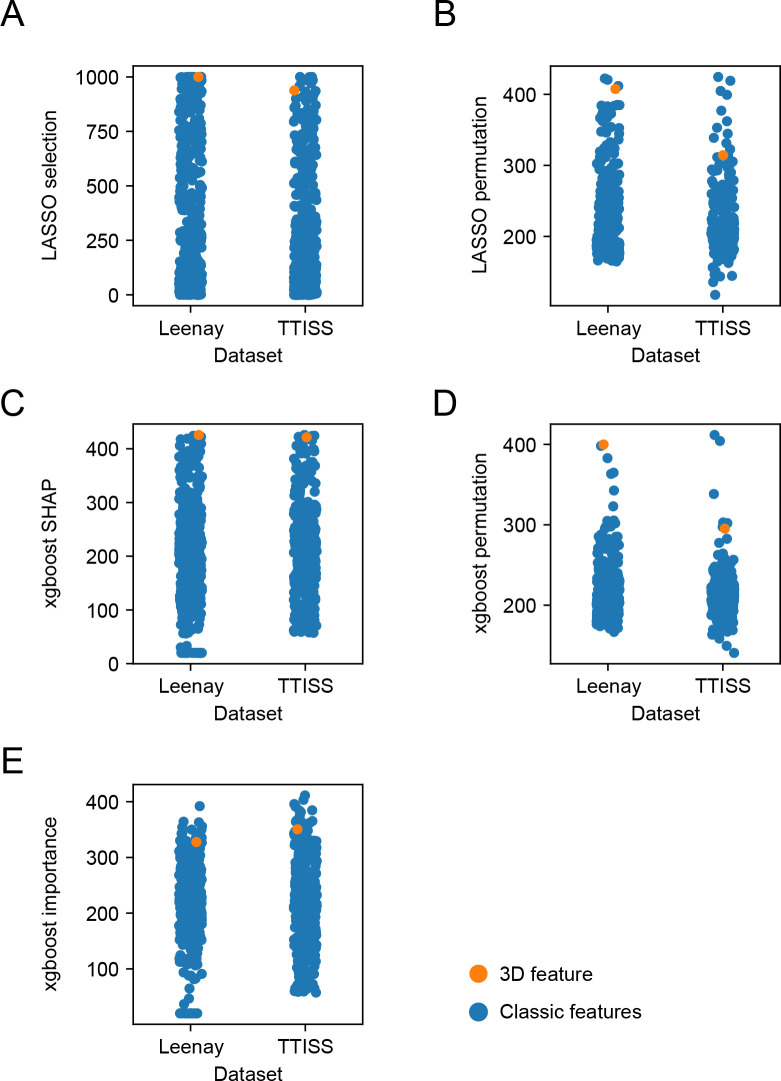
3D features rank highly compared to classic features. Ranks of features used in the LASSO and xgboost models, averaged over the 1000 iterations; a higher rank indicates a more important feature. The 3D feature and classic features are marked with an orange/blue dot, respectively. (A) Number of times each feature was selected in the LASSO models. (B) Feature rank based on LASSO permutation importance. (C) Feature rank based on SHAP values of the xgboost models. (D) Feature rank based on xgboost permutation importance. (E) Feature rank based on xgboost “gain” importance.

For the LASSO model, we counted the number of times each feature was selected during our 1000 iterations (Fig 4A); out of the 426 features, the 3D feature was one of only 7 to be selected in all 1000 models, and one of 35 to be selected more than 950 times in the Leenay dataset. In the TTISS dataset, the 3D feature was the 10th most selected feature, being selected in 93.8% of the models. We also calculated each feature’s permutation importance, calculating the decrease in model performance when shuffling each feature’s values (Fig 4B). In each LASSO iteration, we ranked the features according to this measure and found the average ranking across all 1000 iterations; the 3D feature ranked 4th / 13th in the Leenay / TTISS dataset, respectively.

For the xgboost model, we used the Tree SHAP algorithm to evaluate the features [70]. SHAP is a robust method, originating in game theory, to evaluate and rank the explanative power of predictive features. Similarly to the LASSO permutation ranking, we ranked the features by their SHAP importance in each xgboost iteration and calculated their average rank (Fig 4C); in the Leenay dataset, our 3D feature achieved the highest average rank; on the TTISS dataset, our 3D feature was ranked 5th on average. We repeated the process for xgboost permutation importance (Fig 4D) and xgboost’s built-in “gain” feature importance (Fig 4E); the 3D feature was ranked among the first 20 features in all 4 comparisons. These rankings further establish our 3D features’ importance in both predictive models.

### 3D features could be used to improve predictive models in non-matching cell types

Finally, we wanted to determine whether 3D features generated from data in one cell type can be used to improve CRISPR efficiency prediction in other cell types (i.e. non-matching cell types between the Hi-C data and CRISPR data). To that end, we used 3 Hi-C datasets and 3 CRISPR efficiency datasets. For Hi-C data, we took the T cell and HEK293 datasets previously used, as well as an additional dataset generated from GM12878 cells [71]. For the CRISPR efficiency data, we added the U2OS dataset from GUIDE-Seq [64] to the previously-used Leenay (T cell) and TTISS (HEK293 cells) datasets. We used each set of 3D features on each CRISPR dataset.

**Fig 5:**
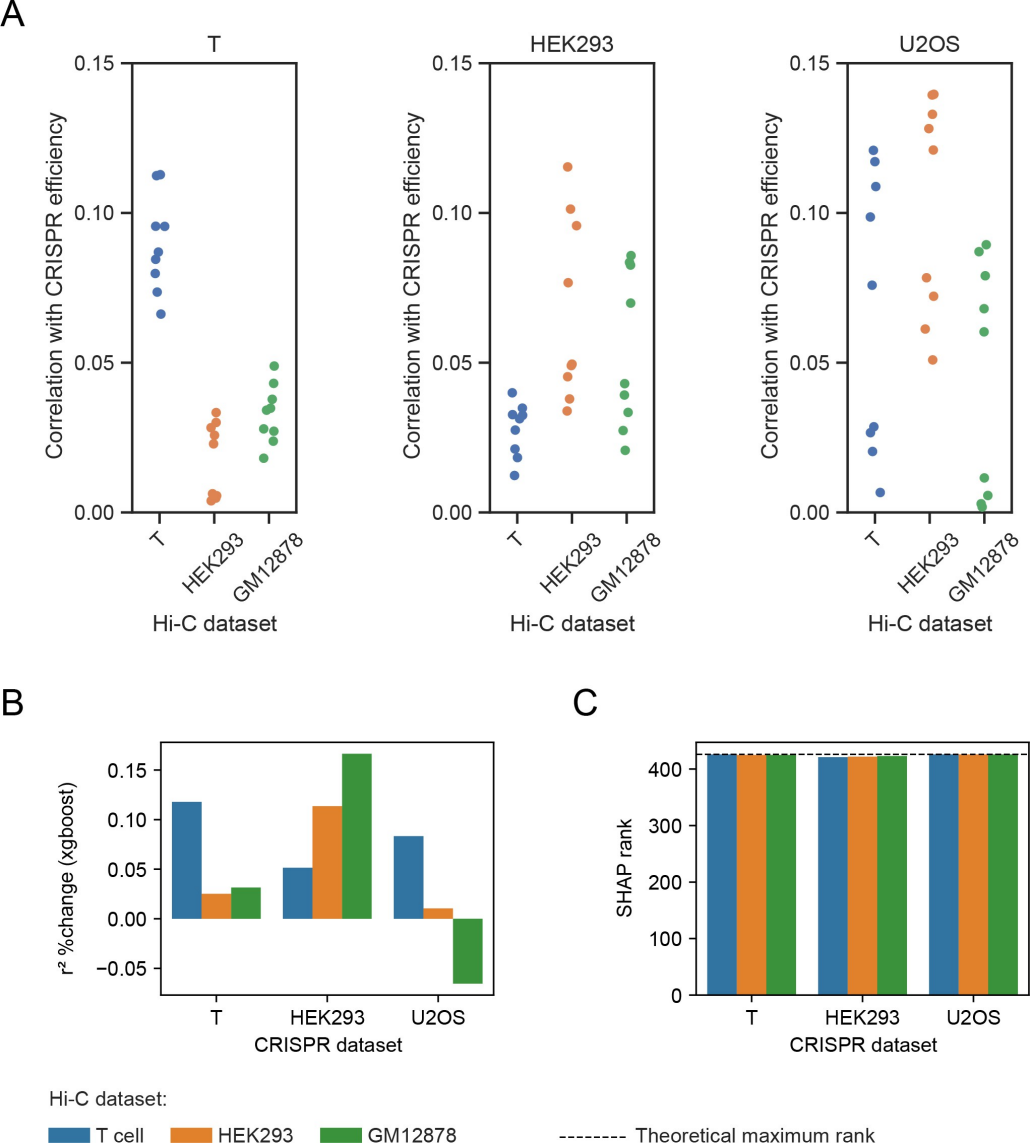
3D features improve prediction in non-matching cell types. (A) Correlations between 3D features, generated from Hi-C in T/HEK293/GM12878 cells, and CRISPR efficiency. (B-C) Blue/orange/green bars represent 3D features generated from Hi-C in T/HEK293/GM12878 cells, respectively. (B) Relative change in xgboost r^2^ before and after adding a 3D feature to the model. (C) Rank of the 3D features relative to the other features, based on their average SHAP rank in the 1000 xgboost iterations; a higher rank is better. The dashed line represents the theoretical rank of a feature ranked first in all 1000 models.

We first compared the correlations of the 3D features with CRISPR efficiency across Hi-C cell types (Fig 5A); in both the HEK293 and U2OS datasets, features from non-matching cell types performed relatively well: in the HEK293 dataset, the best GM12878 feature achieved a correlation of 0.08, compared to a correlation of 0.11 from the best HEK293 feature. For the U2OS dataset we did not have features from U2OS cells, but the correlations of the T cell and HEK293 features were comparable to their correlations on their matching dataset (i.e. T cell features on the Leenay dataset and HEK293 features on the TTISS dataset). In the T cell dataset, non-matching features performed worse than T cell features.

We then conducted the repeated 5-fold cross validation described in the previous section, checking the improvement in prediction power of LASSO and xgboost models when adding a 3D feature. The LASSO models’ predictive ability was not improved when adding a 3D feature based on non-matching cell types, e.g. a HEK293-based 3D feature in the T cell CRISPR dataset (S6A Fig); but the 3D feature was highly selected in the LASSO models (S6B Fig). On the other hand, xgboost models were improved when adding the GM12878 3D feature in the HEK293 CRISPR dataset, and when adding either the T cell or HEK293 3D feature in the U2OS CRISPR dataset (Fig 5B). We note that adding the GM12878 3D feature in the U2OS CRISPR dataset decreased the model improvement, likely due to overfitting on the training set. Similarly to the LASSO models, the 3D features achieved high importance ranks in the xgboost models (Fig 5C). These trends were generally similar when considering the permutation importance and xgboost’s built-in “gain” importance as well (S7 Fig). Thus, in some cases adding a 3D feature may improve the trained model even if the 3D feature was generated from a non-matching cell type.

## Discussion

In this study we evaluated the effect of the 3D structure of the genome on CRISPR efficiency. Despite the need for better computational CRISPR models, and although previous studies have demonstrated the influence of DNA spatial properties on various cellular processes, its relation to CRISPR efficiency was not addressed before.

We calculated 425 well-established CRISPR features, such as the nucleotide composition of the target site and its melting temperature. These features are widely used by existing CRISPR efficiency models and constitute the lion’s share of these models’ features. The best feature out of these 425, in both CRISPR datasets, was a sequence-based feature, which had a correlation of 0.19 / 0.23 / 0.22 with CRISPR efficiency in the Leenay / TTISS / GUIDE-Seq dataset, respectively. The highest correlation of the thermodynamic features was 0.08 / 0.06 / 0.13 (Leenay / TTISS / GUIDE-Seq). The features we generated were highly correlative with CRISPR efficiency relative to the classic features, with the best 3D feature achieving a correlation of 0.11 with CRISPR efficiency, ranking in the top 5% / 13% / 8% correlations out of the whole feature set. In comparison, the average correlation of the classic feature set was 0.04 / 0.06 / 0.06. We note that the best-performing 3D feature differed between the 3 CRISPR datasets; we believe this is a result of specific biases in the Hi-C datasets as well as the CRISPR efficiency datasets. We also note that the CRISPR datasets were generated using completely different protocols, and that the Leenay dataset includes only on-targets, whereas the TTISS and GUIDE-Seq datasets include mainly off-targets. The inconsistency in feature ranking was observed in sequence and thermodynamic features as well, demonstrating the different data patterns between the datasets. These correlations were not decreased when controlling for gene expression levels. Our features uniformly indicated an inverse relation between the 3D properties of the target site and its CRISPR efficiency: sites with lower spatial density demonstrated higher efficiency; this trend was observed in all the features we created.

We compared our features to 5 state-of-the-art models, to demonstrate the value of adding the 3D features, which encode information not included in the current models. Indeed, the 3D features’ correlation with CRISPR efficiency was not decreased when controlling for these models, indicating they added new information.

We also compared our 3D features to 3 epigenetic features; our 3D features’ correlations with CRISPR efficiency remained consistent when controlling for the epigenetic features, indicating they add new information relative to the epigenetic features as well. In addition, the epigenetic features were highly correlative with CRISPR efficiency on the Leenay and GUIDE-Seq datasets, but had very low correlations on the TTISS dataset–whereas the 3D features’ correlation was relatively high in both datasets.

We aimed to check whether adding 3D-based features to a predictive model can improve it; since the state-of-the-art models do not include code to re-train them on new data using new features, we trained new models ourselves. We evaluated the performances of LASSO- and xgboost-based models, before and after adding a single representative 3D feature. Both models performed significantly better after adding the 3D feature, and the 3D feature was ranked among the top 20 features in a variety of feature importance measures (feature selection, SHAP, permutation importance and gain score). The model performance on the Leenay dataset was higher than the model performance on the TTISS dataset (average correlation of 0.3–0.4, as opposed to around 0.2, respectively); this could be due to the nature of the data–the Leenay dataset is based on on-targets, whereas the TTISS dataset is based on off-targets; thus, we would expect this data to be noisier when predicting CRISPR efficiency based on the target sequence and coordinates alone.

We note that while the models we trained seem to outperform the state-of-the-art models, the training and test sets of our Leenay and TTISS models were taken from the same dataset (i.e. Leenay and TTISS, respectively), whereas the 5 state-of-the-art models were trained on other datasets; we would indeed expect models trained on a given dataset to perform better on it. This does not diminish the value of the state-of-the-art models in general; we believe that re-training these models on the datasets used here would improve their performance on them, and that adding our 3D features to them would significantly improve them, since these features contain information that is orthogonal to the models, as we have shown. We believe assessing the state-of-the-art models’ merits on various datasets, while interesting and valuable, is beyond the scope of this study.

We then assessed whether 3D features generated from Hi-C in a given cell type can be used to improve prediction of CRISPR efficiency in other cell types. The results were mixed: in the LASSO models, and in the xgboost model trained on Leenay CRISPR data, the 3D features generated from non-matching cell types did not improve predictive power; while they did improve xgboost models trained on HEK293 and U2OS CRISPR datasets. Thus, we believe the 3D features should be considered for potential use in models on any endogenous CRISPR dataset, even if generated from a different cell type, although the predictive power they provide needs to be assessed based on the specific model and training/test data. In addition to our explicit analysis, we note that many aspects of 3D organization are conserved between different tissues and organisms [67,72–75]–thus, even 3D features from other cell types may improve CRISPR prediction.

These results imply the existence of novel information relative to the tested models: existing models use mainly local sequence and thermodynamic features to predict CRISPR efficiency, but the 3D structure of the genome includes information that (a) is not contained in these features, and (b) can improve existing predictive tools. More and more studies demonstrate the relation between the genomic 3D structure and various biological processes, such as tissue development [76] and gene expression [66,67,77,78]. The spatial conformation of DNA dictates distance between genomic regions, which enables the interaction and co-regulation of regions that are closer together, while other regions could be sequestered to reduce potential interactions.

**Fig 6:**
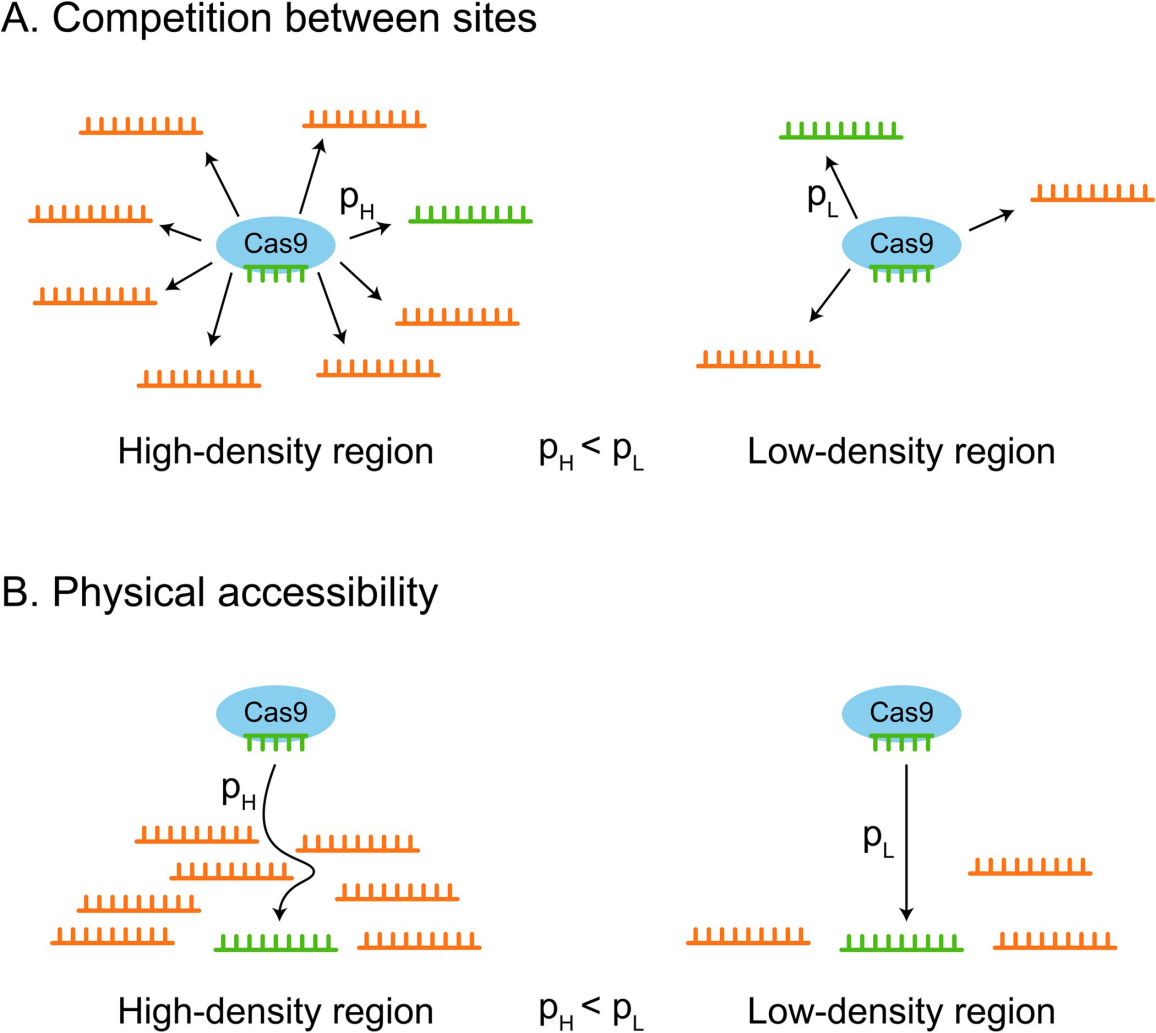
Hypotheses explaining the inverse relation between genomic 3D density and CRISPR efficiency. Unsuitable target sites are colored in orange, and suitable, fully complementary target sites are colored in green. p_H_/p_L_ is the probability of binding to the suitable target site in the high-density/low-density region, respectively. (A) A Cas9-sgRNA complex searching for a target site in a dense region (left) encounters many unsuitable target sites; thus, the probability of finding the suitable target site in the dense region is lower than in a low-density region (right), where significantly fewer potential target sites exist. (B) Target sites in high-density regions (left) are less accessible physically to the Cas9-sgRNA complex, relative to low-density regions (right), because of DNA packing in the region.

The sgRNA-Cas complex searches for a suitable target site in a stochastic manner via three-dimensional diffusion, sampling several target sites for complementarity [79,80]. We propose that tightly packed regions could overload the CRISPR machinery with a large number of non-complementary target sites, forcing the sampling of more sites and lowering the probability of encountering a suitable one (Fig 6A). The initial association between the CRISPR complex and DNA occurs through PAM (protospacer-adjacent motif) recognition [79,81,82]; despite the speediness of dissociation from a non-suitable site [80], a dense genomic region would provide an abundance of potential PAMs (e.g. almost any “GG” dinucleotide, in the case of SpCas9), significantly slowing the process. This is in accordance with the consistent inverse relation we have observed between 3D density and CRISPR efficiency.

Another possibility is that denser regions may be less physically accessible to the CRISPR complex (Fig 6B). Previous studies have shown chromatin accessibility to be correlated with CRISPR efficiency [83–86], these studies have dealt with nucleosome occupancy, e.g. MNase-seq and ATAC-seq data, rather than the global 3D structure of each chromosome via Hi-C data. Both of these “accessibilities”–the interactions-based (our study) and the nucleosome-based (previous studies)–could be useful and informative in their own right, as we demonstrated in our comparison between epigenetic features and 3D features. Even when the site is physically accessible, the Hi-C based features encode the number of potential targets around the target site, whereas the epigenetic features contain this information to a lesser extent. Finally, the relation between 3D density and CRISPR efficiency may be indirect, through other elements that are related both to 3D density and CRISPR efficiency. These are interesting and valuable directions for further research and empirical validation.

Based on this study’s results, we believe the combination of local features (the properties of the specific 20nt target site and its immediate surroundings) with global features (e.g. structural properties of the genome, in the scale of 1-1000kb) would provide the best predictive model for CRISPR efficiency.

The main limitations of our work have to do with sparsity of data, both from the 3D and CRISPR ends: a desirable 3D dataset would be high-resolution (≤10kb) Hi-C, to differentiate between neighboring target sites; few such datasets exist. In addition, the CRISPR efficiency dataset–which ideally would be measured in the same cell type as the Hi-C data–needs to be measured in-vivo and include data regarding the genomic location of the target sites. The CRISPR datasets we have used are the largest ones to comply with these requirements. Thus, the information contained in the 3D structure of the genome may be even higher than demonstrated in this study; and future work, based on more comprehensive datasets–both for in-vivo CRISPR efficiency and Hi-C data–may yield higher correlations between the two. We also note that Hi-C measurements, based on cleavage and digestion assays, may be biased to encode similar information to CRISPR assays, which include cleavage and digestion as well. While we cannot fully rule out the existence of such biases in Hi-C, we note that this concern is shared by all features based on DNA cleavage and digestion, which include the well-established epigenetic features based on DNAse-Seq and ChIP-Seq. We believe that the steps we took to smooth the biases in the data, by normalizing interaction frequencies and using graph paths instead of a single interaction frequency to estimate the pairwise 3D distances, alleviate this concern enough to consider using 3D features as predictive and informative features. We note that in addition to these steps, we control for dozens of other features, demonstrating that our features remain significant; this supports the conjecture that the 3D features’ signal is at least partially not due to confounding factors.

When considering global features that affect CRISPR efficiency–as they will in real-world in-vivo applications of CRISPR–the CRISPR data should, ideally, be measured in as natural an environment as possible, i.e. insertion of CRISPR complexes into cells. Thus, when predicting CRISPR’s action, the cell type and the nature of the data should be considered. This is especially true with global features, which highly depend on the cell type (e.g. the 3D structure of a T cell would be different than the structure of a HeLa cell).

We have demonstrated the correlative relation between the 3D structure of the genome and CRISPR; but there remains much to be discovered regarding this relation. One way to pursue this direction is to design gRNAs with multiple known target sites in both high-density and low-density genomic regions; the editing efficiencies at these sites could then be compared, to see whether the same target sequence in the low-density region is a significantly more efficient target site than the high-density one. Other questions arise as well–is there a direct causal relation between CRISPR efficiency and 3D structure, or can this relation be attributed to a third determinant? On what genomic scale can this relation be observed? As more in-situ CRISPR and high-resolution Hi-C data are published, we will be able to better understand the relation between the two; provide insight into CRISPR’s mechanism; and, ultimately, improve our ability to predict CRISPR cleavage, as well as design sgRNAs for therapeutic and scientific use.

## Materials and methods

### Generating a chromosomal 3D structure based on Hi-C reads

We downloaded per-chromosome matrices of interaction frequencies (*IF*) based on Hi-C reads generated in T cells [60], HEK293 [61] and GM12878 [71]. We used Juicer [87] to extract the normalized interaction matrices available in the “hic” files (KR normalization for the T cell dataset, and VC normalization for the HEK293 and GM12878 datasets). Since the *IF* of two genomic bins is inversely proportional to their 3D distance (*d*), we modeled the relationship as $d=\frac{1}{IF}$ (S8A Fig). We then represented each chromosome as a weighted graph, where each node is a 10kb genomic region, and the weight of each edge is the distance *d* between the two nodes, as defined above.

Next, for each pair of nodes, we found all possible graph paths consisting of up 2 to edges between these nodes, and calculated each path’s length as the sum of its edges’ weights. We defined the distance between two nodes as the average length of the 5 shortest such paths. We chose this method to reduce the noise in the measurements and acquire a characteristic distance between the two nodes, taking into account feasible routes. We have initially tried to simply use the shortest possible path between the two nodes, without considering the number of edges it includes, but this approach did not produce satisfactory results, with correlations of ~0.01 between our features and the CRISPR efficiency data.

### Calculating 3D-based features

Having estimated all bin-bin distances for each chromosome, we calculated two sets of features for each bin B:

For the Distance (n) features (S8B Fig), we sorted the distances between bin B and each other bin in the chromosome, and then calculated the average of the n shortest distances (*n*∈1000,2500,5000,10000).

For the Radius (m) features (S8C Fig), we first determined a cutoff relative to all distances in the chromosome: we sorted all N bin-bin distances in a vector (where position 1 contains the shortest distance and position N contains the longest distance) and took as a radius the distance at position $N∙\frac{m}{100}(m\in 10,15,20,25,30)$; we denoted this distance r. We then counted the number of bins whose distance from B is less than or equal to r.

The code used to generate these features is available in https://github.com/aescrdni/3density.

### Calculating TAD and A/B compartment features

We used the Arrowhead module in Juicer [87] to extract TAD coordinates in each Hi-C dataset, with a resolution of 100kb. For each site, we calculated two TAD features: whether it resided inside a TAD (1 if so, 0 otherwise); and the TAD’s length. For A/B compartment calling, we used Juicer Eigenvector, with a resolution of 1Mb; each site’s A/B feature was the sign of its genomic region’s value in the eigenvector.

### Calculating “classic” and epigenetic features

We calculated 428 features overall: 420 sequence-based features, 5 thermodynamic features and 3 epigenetic features (S2 and S3 Tables).

The sequence-based features included: (A) binary features denoting the identity of the nucleotide at each position (4nt × 20 positions = 80 features); (B) binary features denoting the identity of the dinucleotide at each pair of positions (16 × 20 = 320 features); (C) the overall frequency of each nucleotide (4 features); and (D) the overall frequency of each dinucleotide (16 features).

The thermodynamic features are similar to the ones calculated in [8]: we calculated the free energy of the sgRNA (using RNAfold [88]), and the melting temperatures (using Biopython) of 4 regions along the target site (positions 1–20, 1–4, 5–12 and 16–20).

The epigenetic features assessed the accessibility and activity of the target site’s DNA region, based on DNase-Seq, TF ChIP-seq and Histone ChIP-seq data downloaded from ENCODE [89]. Details regarding the data appear in the S1 Text and S2 and S3 Tables.

All the figures’ underlying data appears in S6 Table.

## Supporting information

S1 Text Supplementary methods. (DOCX)

S1 Table 3D feature values. The “Distance (2500)” (T cell), “Distance (1000)” (HEK293) and “Radius (10)” (HEK293) feature values of all human (hg19) genomic bins, in 10kb resolution. Bins whose feature value is 0 were omitted from the table.
(XLSX)

S2 Table The "classic" features used in our model. (XLSX)

S3 Table ENCODE data used in the study. (XLSX)

S4 Table Features included in the 5 state-of-the-art models. (XLSX)

S5 Table Average feature ranks in the LASSO and xgboost models. The average rank of the 3D feature’s importance value over the 1000 cross-validation iterations described in the paper. A lower rank denotes a more informative feature.
(XLSX)

S6 Table The manuscript figures’ underlying numerical data. (XLSX)

S1 Fig Correlations between CRISPR efficiency and 3D density at different Hi-C resolutions. Strip plots of Pearson correlations between CRISPR efficiency measurements and 9 3D density features, generated from 3 different Hi-C resolutions (10kb, 25kb and 50kb); as well as 2 TAD features in 100kb resolution and one A/B compartment feature in 1Mb resolution.
(TIF)

S2 Fig Scatter plot of CRISPR efficiency vs 3D distance. Scatterplots of CRISPR efficiency measurements vs. a representative 3D feature, for the Leenay dataset (1574 observations), TTISS dataset (666 observations) and GUIDE-Seq dataset (153 observations).
(TIF)

S3 Fig 3D density is inversely proportional to CRISPR efficiency. Boxplots of the “Distance” and “Radius” features, for the sites with top/bottom 20% efficiency (orange/blue plots, respectively) in the (A) Leenay dataset, (B) TTISS dataset and (C) GUIDE-Seq dataset. Significance of difference between the top and bottom sites was calculated using Wilcoxon’s rank-sum test. * p < 0.05; ** p < 0.01; *** p < 0.001
(TIF)

S4 Fig 3D features reduce model error. Histograms of the RMSE of the models trained on the Leenay and TTISS datasets, when testing LASSO and xgboost models. The blue/orange histogram indicates the model with/without the 3D feature, respectively. p-values were calculated using Wilcoxon’s signed rank test. (A) Models using all 425 classic features; (B) Models using the top 30 features, based on Pearson correlation with CRISPR efficiency.
(TIF)

S5 Fig Model performance on the GUIDE-Seq (HEK293) dataset. Histograms of the correlations between measured and predicted efficiency in the GUIDE-Seq (HEK293) dataset, when testing LASSO and xgboost models. The blue/orange histogram indicates the model with/without the 3D feature and the average correlation is marked with a solid/dashed line, respectively. (A) Models using all 425 classic features; (B) Models using the top 30 features, based on Pearson correlation with CRISR efficiency.
(TIF)

S6 Fig LASSO performance when using non-matching cell types. Blue/orange/green bars represent 3D features generated from Hi-C in T/HEK293/GM12878 cells, respectively. (A) Relative change in LASSO r^2^ before and after adding a 3D feature to the model. (B) Rank of the 3D features relative to the other features, based on number of selections in the 1000 LASSO iterations. The dashed line represents theoretical rank of a feature selected in all 1000 models. A higher rank is better.
(TIF)

S7 Fig 3D features rank highly in non-matching cell types. Average importance ranks of the 3D features compared to other features, over 1000 model iterations. Blue/orange/green bars represent 3D features generated from Hi-C in T/HEK293/GM12878 cells, respectively. The dashed line represents the theoretical rank of a feature ranked first in all 1000 models. (A) Permutation importance in LASSO models. (B) Permutation importance in xgboost models. (C) Gain importance in xgboost models.
(TIF)

S8 Fig Generating 3D features based on Hi-C data. (A) The pipeline for estimating inter-chromosomal distances. We converted each chromosomal interaction matrix (left) to a weighted graph (center), where each node is a 10kb genomic region, and an edge represents an interaction between two regions. The weight of each edge is 1 / (number of interactions). Next, for each pair of regions on the same chromosome (green nodes), we found the p shortest paths between them (dashed edges) and took the distance between them to be the average weight of these paths (right). (B) An illustration of the “Distance (n)” feature–for a given genomic region (highlighted blue), we found the n closest regions (green), and calculated their average distance from it. (C) An illustration of the “Radius (m)” feature–for a given genomic region (highlighted blue), we counted the number of nodes (green) closer to it than a certain radius. The radius was determined relatively to all the inter-chromosomal distances found (e.g. the distance in the top 10%).
(TIF)
