# Supplementary material for: Strong association between genomic 3D structure and CRISPR cleavage efficiency
Source: PLoS Comput Biol. 2024 Jun 7;20(6):e1012214. doi: 10.1371/journal.pcbi.1012214 (PMC11189236; doi:10.1371/journal.pcbi.1012214)
Supplement: S1 Text — (DOCX) [file pcbi.1012214.s001.docx]

**Supplementary material for “Strong association between genomic 3D structure and CRISPR cleavage efficiency”**

**Results**

**Model training in the GUIDE-Seq (HEK293) dataset**

We repeated the analysis described in the Results subsection “Adding a 3D feature significantly improves CRISPR predictive power” on the GUIDE-Seq dataset, using 200 repeats of 3-fold cross validation; we chose 3-fold cross validation since using 5-fold cross validation, as we did in the previous cases, would result in a very small validation set (n = 31). Both the LASSO and xgboost models were highly overfit, even when using the reduced set of features, and achieved average correlations of around -0.01 (S5 Fig). We believe this is due to the small size of the dataset; the training set includes 102 observations, whereas the training sets for the Leenay and TTISS datasets included more than 500 observations (which is larger than the whole GUIDE-Seq HEK293 dataset). Similar results were observed when using 5-fold cross validation.

**Methods**

**Acquiring CRISPR efficiency data**

The Leenay dataset, generated in T cells, was published in (1). In that study, 1521 unique gRNAs were designed to affect 1656 unique on-targets. The gRNAs were combined with Cas9, and the CRISPR complexes were electroporated into T cells; thus the CRISPR cleavage was conducted in-vivo and on endogenous target sites. The on-targets underwent targeted sequencing, based on which the authors generated a repair profile for each site. We downloaded the “MutationEfficiency”, “IndelCounts” and “Insertion” files from the study’s supplementary data; taking only sites with at least 1000 mapped reads, we defined the CRISPR efficiency of a given site to be the fraction of edited reads out of the total number of the reads mapped to that site. This method yielded 1574 efficiency values for CRISPR on-targets.

The TTISS dataset was generated in HEK293 (2) by the GUIDE-Seq protocol (3): inserting tags into DSBs, sequencing the genome to find these tags and quantifying the number of mapped reads, assumed to be indicative of CRISPR activity at the site. This protocol was conducted in-vivo as well. We took the average number of SpCas9 Seq reads as efficiency scores, keeping only sites with at least one such read. This resulted in using 666 of the 689 target sites.

The GUIDE-Seq dataset (3), included 260 sites in U2OS cells and 153 sites in HEK293. We took the number of GUIDE-Seq reads as efficiency scores.

**Acquiring gene expression levels**

We downloaded normalized expression levels from the Expression Atlas website (4) (T cell: accession number E-GEOD-36765, HEK293: accession number E-GEOD-14429), which were generated from the data published in (5) and (6). To find whether target sites reside in genes, we used the Ensembl v.101 annotation (7). For each target site, we checked whether its coordinates overlap with the coding sequence of any gene on the same chromosomal strand by at least one position.

**Acquiring epigenetic data**

We downloaded bigwig files from the ENCODE project (8) including information regarding DNase-seq, TF ChIP-seq (CTCF) and Histone ChIP-seq (H3K4me3). We calculated the epigenetic features by averaging the bigwig values over the target site’s coordinates. Full details appear in Tables S1-2.

**Calculating feature importance using SHAP values**

To calculate the importance of the 426 features used in our xgboost models, we used the TreeExplainer algorithm from the SHAP (SHapley Additive exPlanations) python package (9). SHAP values improve the interpretability of gradient-boosted models (and tree models in general) by estimating the additive contribution of each feature in the model. Briefly, for each individual observation in the dataset, the SHAP value of a given feature is the additive change it induces relative to the expected model prediction, i.e. how much it increases or decreases the expected prediction to arrive at the actual prediction of the model for the given observation. Thus, a SHAP value of higher magnitude (either positive or negative) indicates a higher influence of the feature on the model’s prediction, i.e. higher feature importance. From this local, per-observation interpretability, we can arrive at a global, per-model interpretability, by averaging over the absolute values of all per-observation SHAP values for a given feature.

**References**

1. Leenay RT, Aghazadeh A, Hiatt J, Tse D, Roth TL, Apathy R, et al. Large dataset enables prediction of repair after CRISPR–Cas9 editing in primary T cells. Nat Biotechnol [Internet]. 2019;37(9):1034–7. Available from: https://doi.org/10.1038/s41587-019-0203-2

2. Schmid-Burgk JL, Gao L, Li D, Gardner Z, Strecker J, Lash B, et al. Highly Parallel Profiling of Cas9 Variant Specificity. Mol Cell [Internet]. 2020;78(4):794-800.e8. Available from: https://www.sciencedirect.com/science/article/pii/S109727652030143X

3. Tsai SQ, Zheng Z, Nguyen NT, Liebers M, Topkar V V, Thapar V, et al. GUIDE-seq enables genome-wide profiling of off-target cleavage by CRISPR-Cas nucleases. Nat Biotechnol [Internet]. 2015;33(2):187–97. Available from: https://doi.org/10.1038/nbt.3117

4. Papatheodorou I, Moreno P, Manning J, Fuentes AM-P, George N, Fexova S, et al. Expression Atlas update: from tissues to single cells. Nucleic Acids Res [Internet]. 2020 Jan 8;48(D1):D77–83. Available from: https://doi.org/10.1093/nar/gkz947

5. Gu-Trantien C, Loi S, Garaud S, Equeter C, Libin M, de Wind A, et al. CD4+ follicular helper T cell infiltration predicts breast cancer survival. J Clin Invest [Internet]. 2013 Jul 1;123(7):2873–92. Available from: https://doi.org/10.1172/JCI67428

6. Chang AHK, Jeong J, Levine RL. Iron Regulatory Protein 2 Turnover through a Nonproteasomal Pathway*. J Biol Chem [Internet]. 2011;286(27):23698–707. Available from: https://www.sciencedirect.com/science/article/pii/S0021925819487206

7. Cunningham F, Allen JE, Allen J, Alvarez-Jarreta J, Amode MR, Armean IM, et al. Ensembl 2022. Nucleic Acids Res [Internet]. 2022 Jan 7;50(D1):D988–95. Available from: https://doi.org/10.1093/nar/gkab1049

8. Luo Y, Hitz BC, Gabdank I, Hilton JA, Kagda MS, Lam B, et al. New developments on the Encyclopedia of DNA Elements (ENCODE) data portal. Nucleic Acids Res [Internet]. 2020 Jan 8;48(D1):D882–9. Available from: https://doi.org/10.1093/nar/gkz1062

9. Lundberg SM, Erion G, Chen H, DeGrave A, Prutkin JM, Nair B, et al. From local explanations to global understanding with explainable AI for trees. Nat Mach Intell [Internet]. 2020;2(1):56–67. Available from: https://doi.org/10.1038/s42256-019-0138-9
