# Supplementary figures and images for: Strong association between genomic 3D structure and CRISPR cleavage efficiency
Source: PLoS Comput Biol. 2024 Jun 7;20(6):e1012214. doi: 10.1371/journal.pcbi.1012214 (PMC11189236; doi:10.1371/journal.pcbi.1012214)

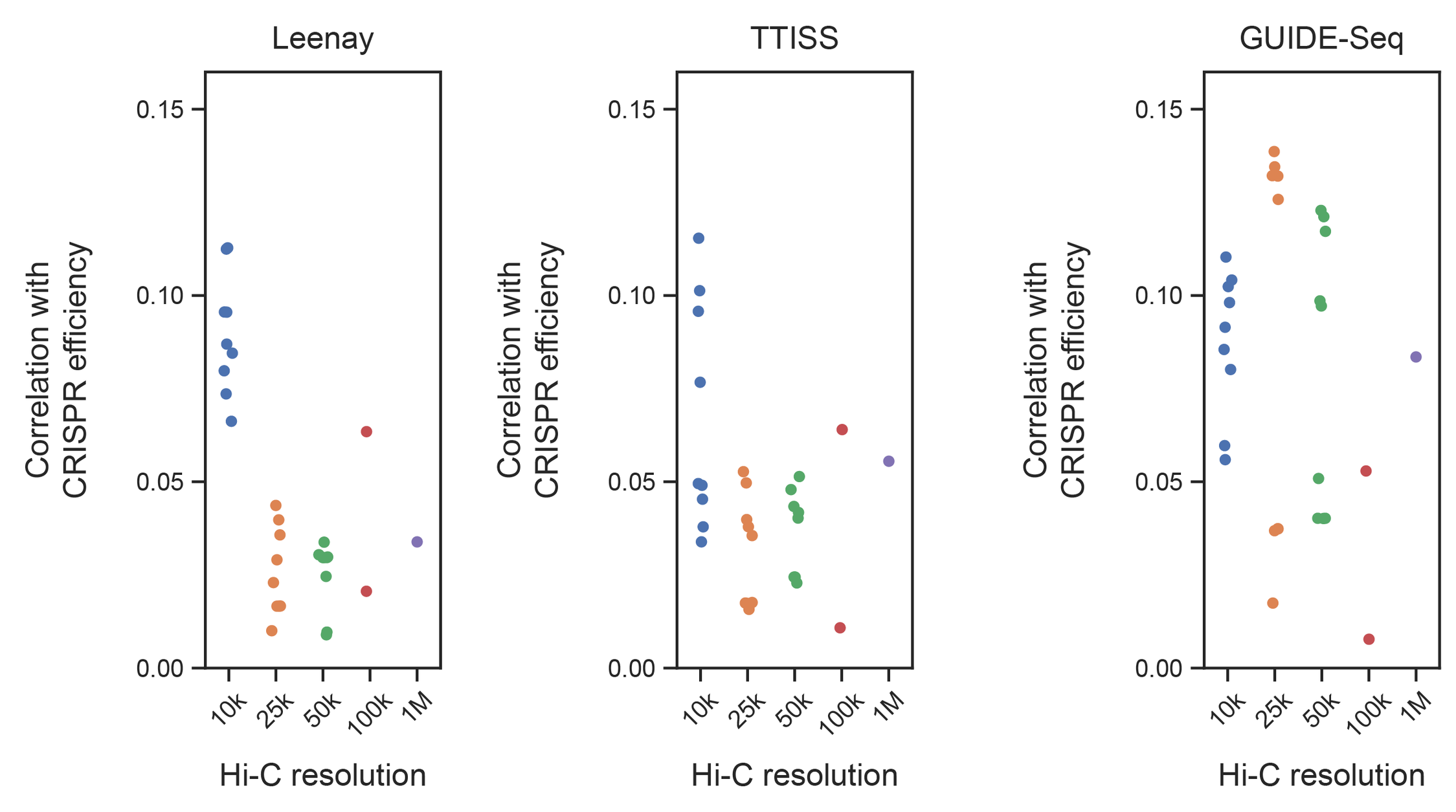

Supplement: S1 Fig — Strip plots of Pearson correlations between CRISPR efficiency measurements and 9 3D density features, generated from 3 different Hi-C resolutions (10kb, 25kb and 50kb); as well as 2 TAD features in 100kb resolution and one A/B compartment feature in 1Mb resolution. (TIF) [file pcbi.1012214.s008.tif]

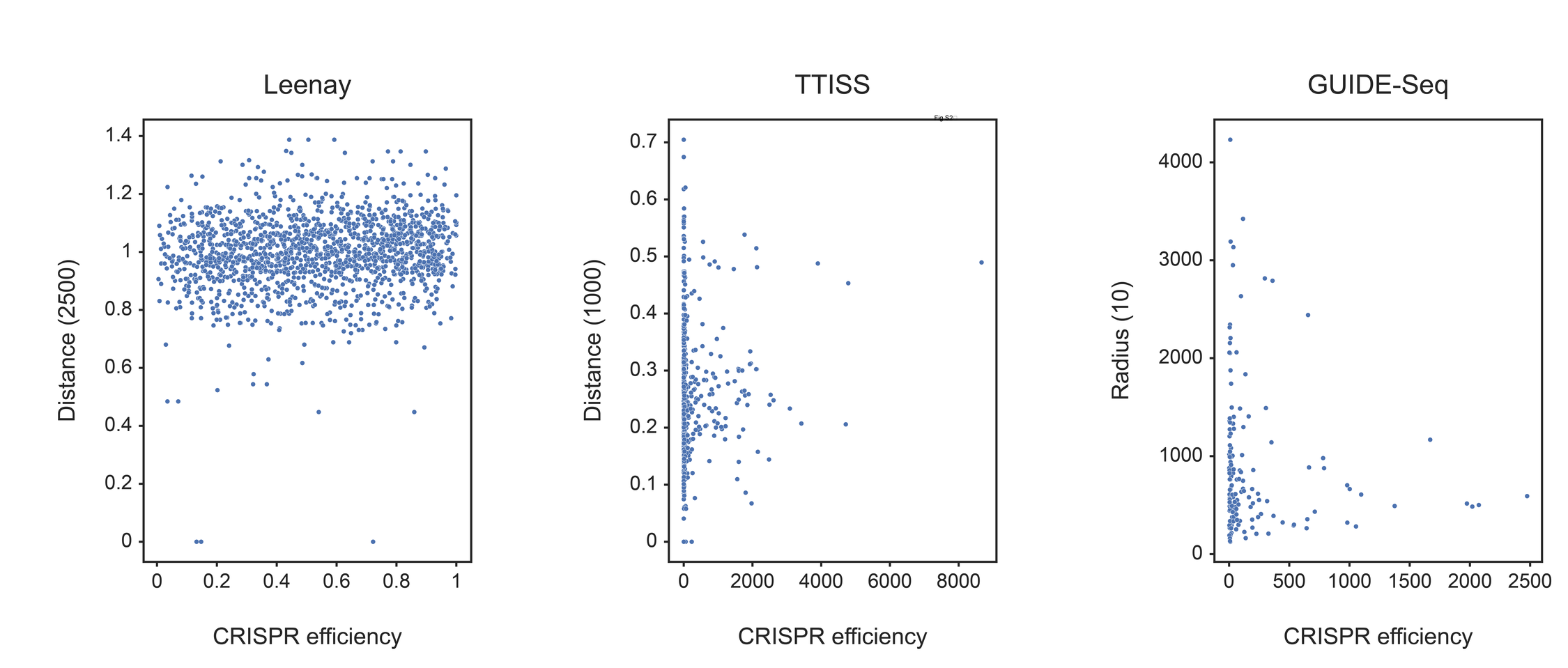

Supplement: S2 Fig — Scatterplots of CRISPR efficiency measurements vs. a representative 3D feature, for the Leenay dataset (1574 observations), TTISS dataset (666 observations) and GUIDE-Seq dataset (153 observations). (TIF) [file pcbi.1012214.s009.tif]

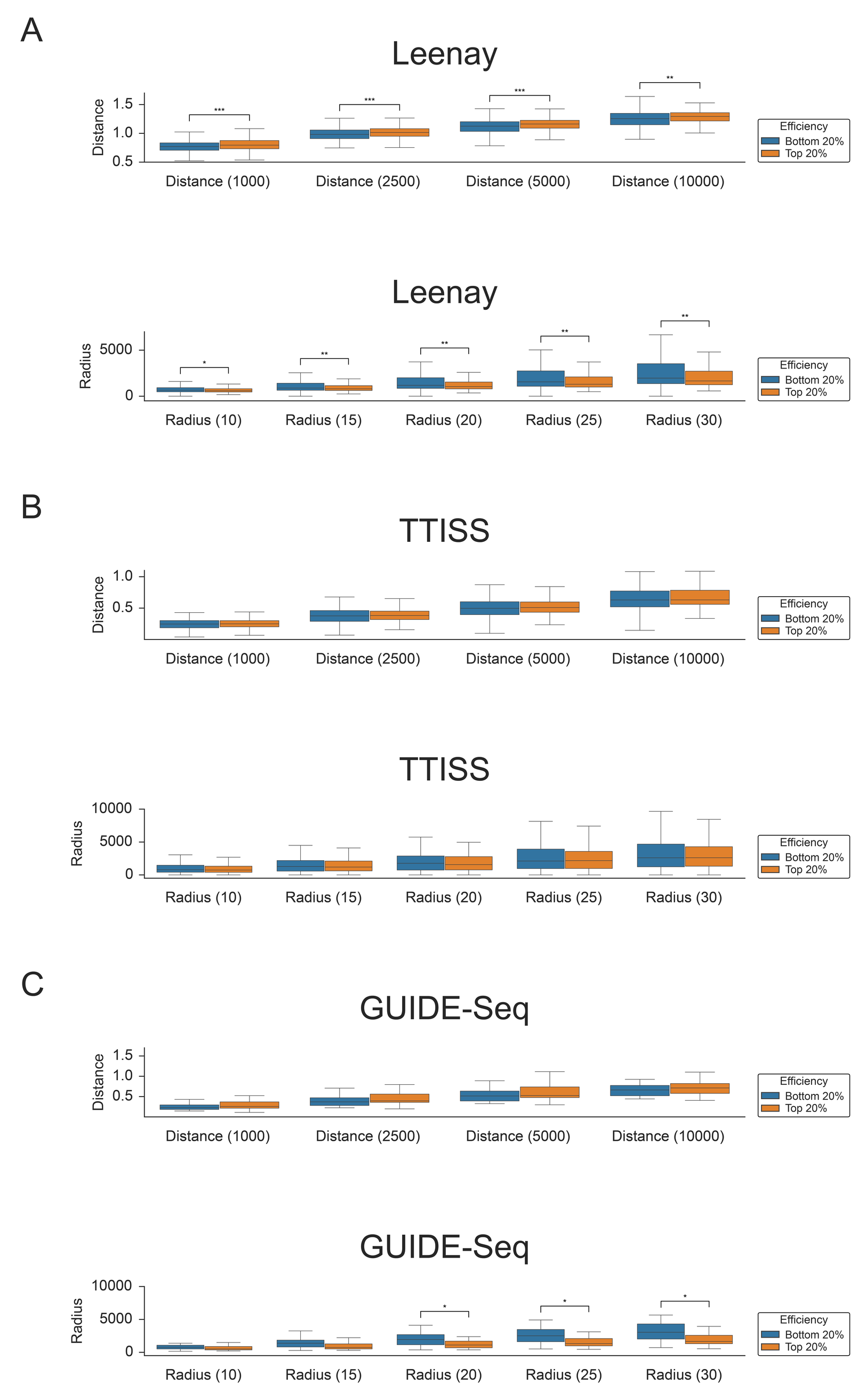

Supplement: S3 Fig — Boxplots of the “Distance” and “Radius” features, for the sites with top/bottom 20% efficiency (orange/blue plots, respectively) in the (A) Leenay dataset, (B) TTISS dataset and (C) GUIDE-Seq dataset. Significance of difference between the top and bottom sites was calculated using Wilcoxon’s rank-sum test. * p < 0.05; ** p < 0.01; *** p < 0.001 (TIF) [file pcbi.1012214.s010.tif]

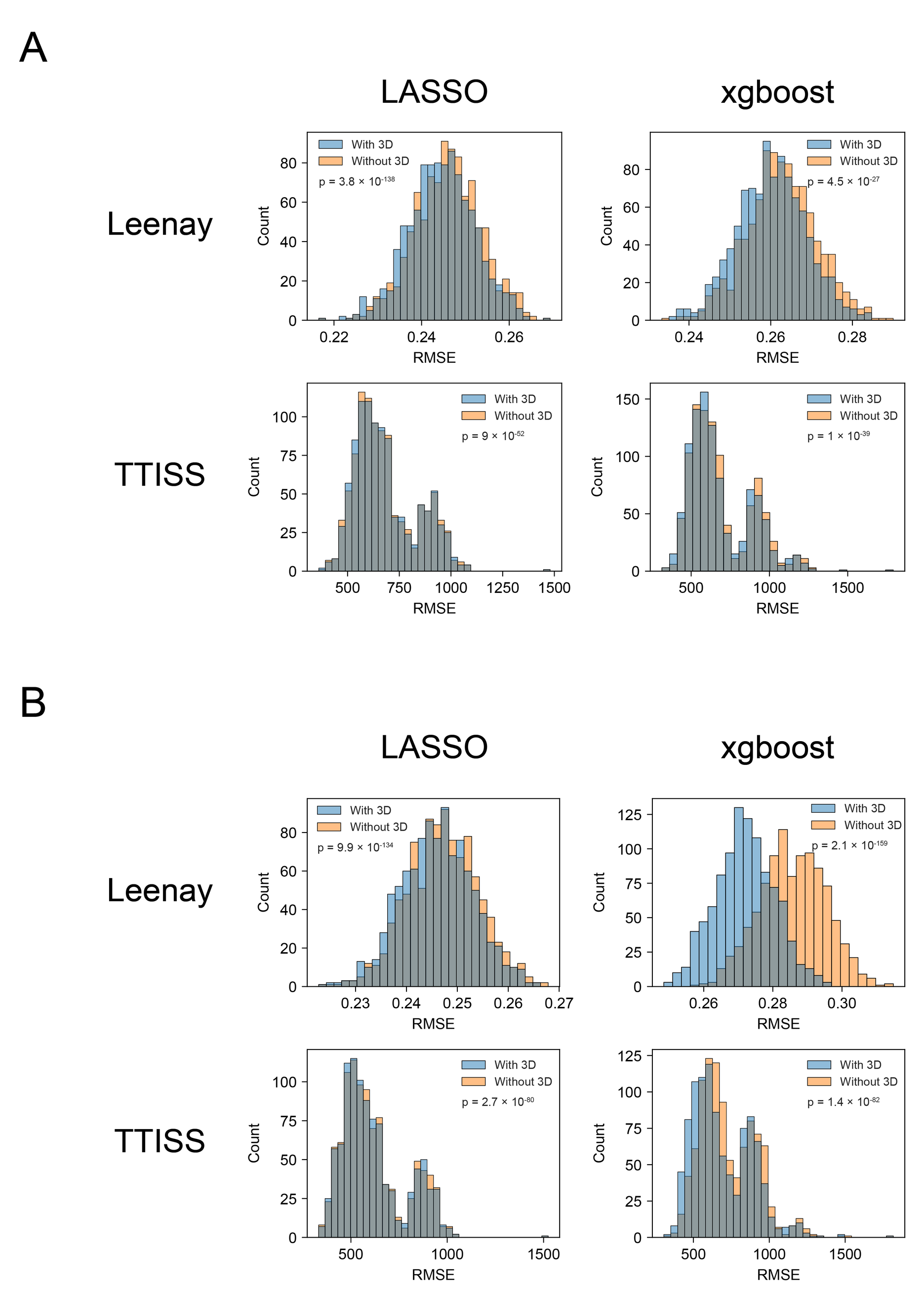

Supplement: S4 Fig — Histograms of the RMSE of the models trained on the Leenay and TTISS datasets, when testing LASSO and xgboost models. The blue/orange histogram indicates the model with/without the 3D feature, respectively. p-values were calculated using Wilcoxon’s signed rank test. (A) Models using all 425 classic features; (B) Models using the top 30 features, based on Pearson correlation with CRISPR efficiency. (TIF) [file pcbi.1012214.s011.tif]

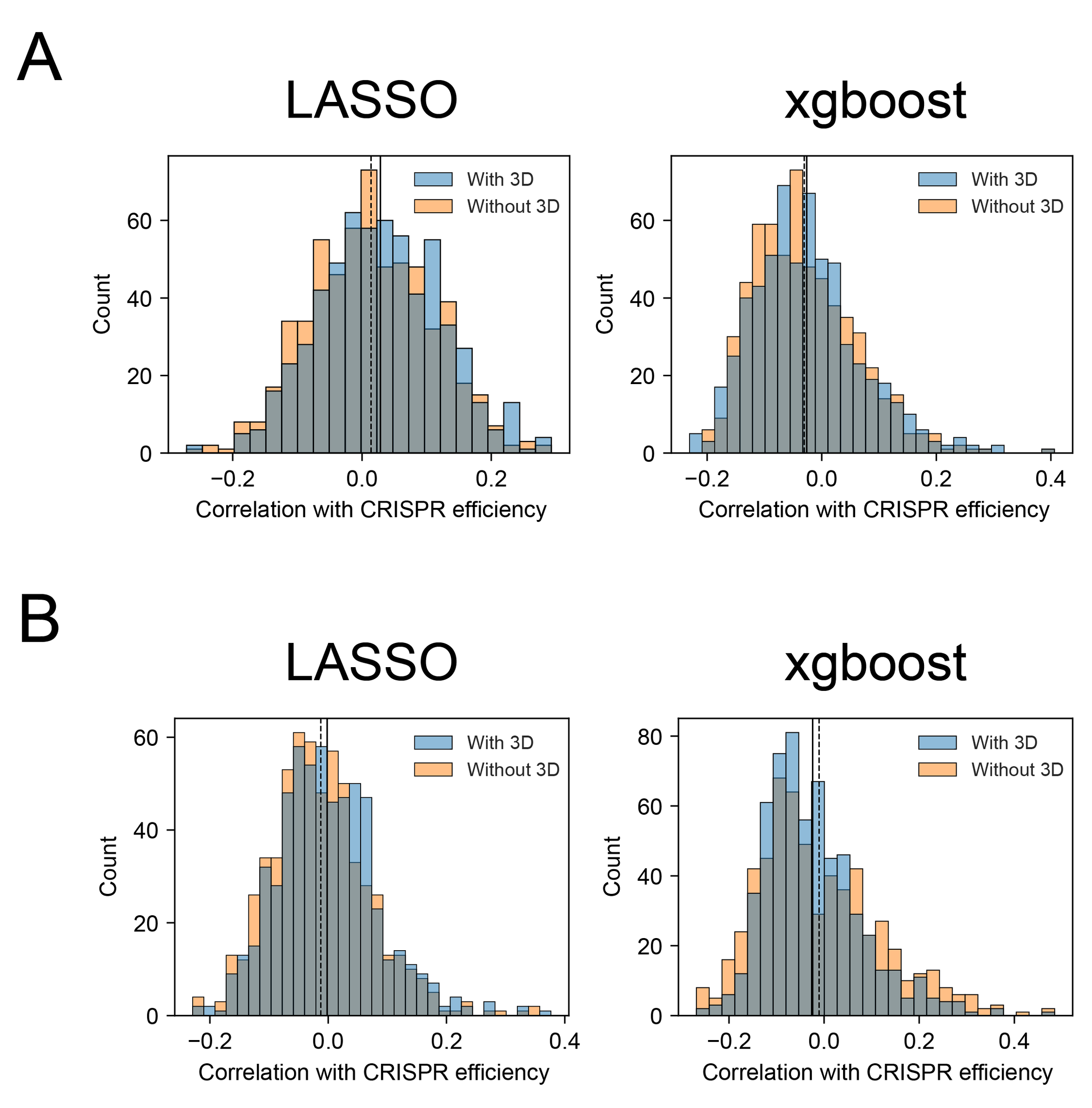

Supplement: S5 Fig — Histograms of the correlations between measured and predicted efficiency in the GUIDE-Seq (HEK293) dataset, when testing LASSO and xgboost models. The blue/orange histogram indicates the model with/without the 3D feature and the average correlation is marked with a solid/dashed line, respectively. (A) Models using all 425 classic features; (B) Models using the top 30 features, based on Pearson correlation with CRISR efficiency. (TIF) [file pcbi.1012214.s012.tif]

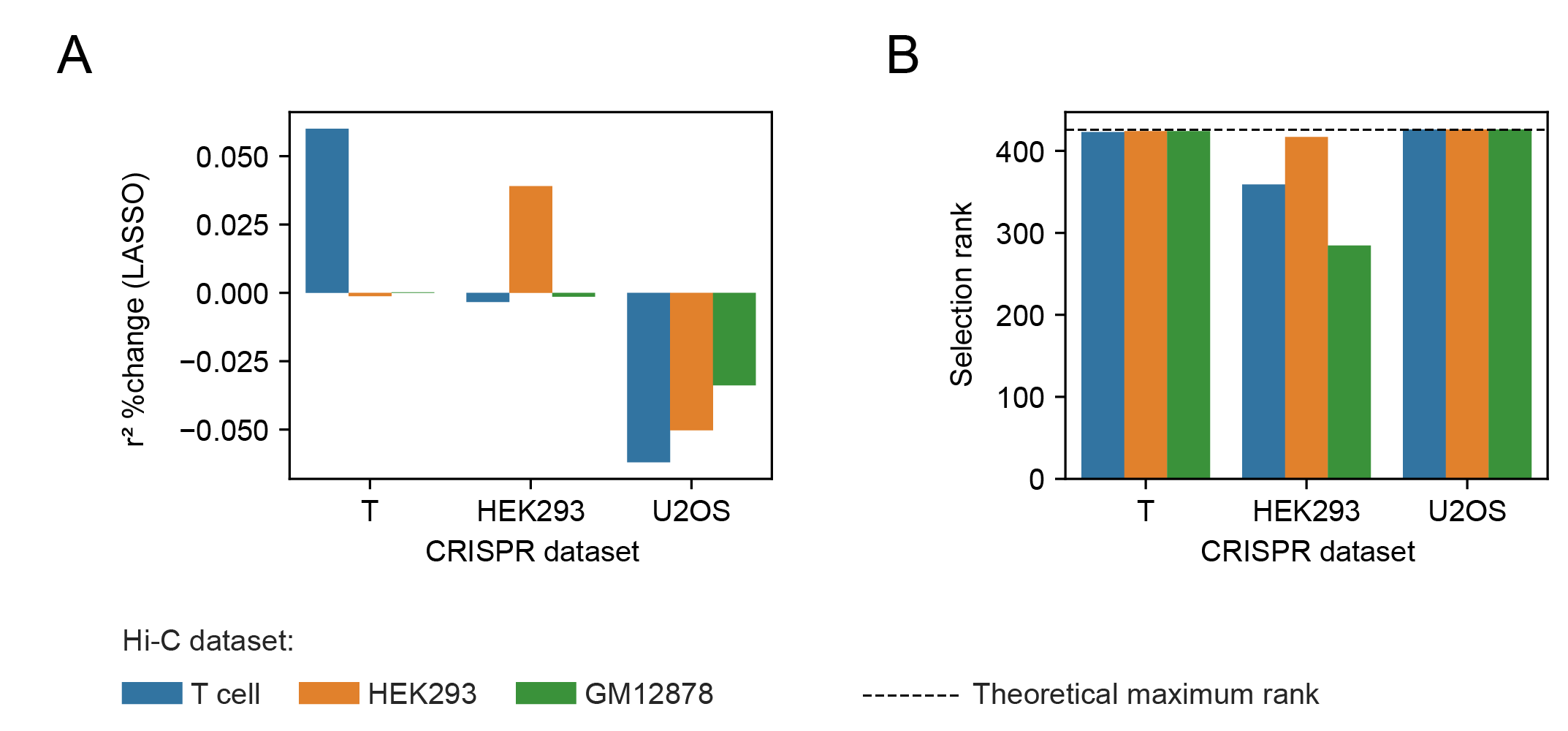

Supplement: S6 Fig — Blue/orange/green bars represent 3D features generated from Hi-C in T/HEK293/GM12878 cells, respectively. (A) Relative change in LASSO r2 before and after adding a 3D feature to the model. (B) Rank of the 3D features relative to the other features, based on number of selections in the 1000 LASSO iterations. The dashed line represents theoretical rank of a feature selected in all 1000 models. A higher rank is better. (TIF) [file pcbi.1012214.s013.tif]

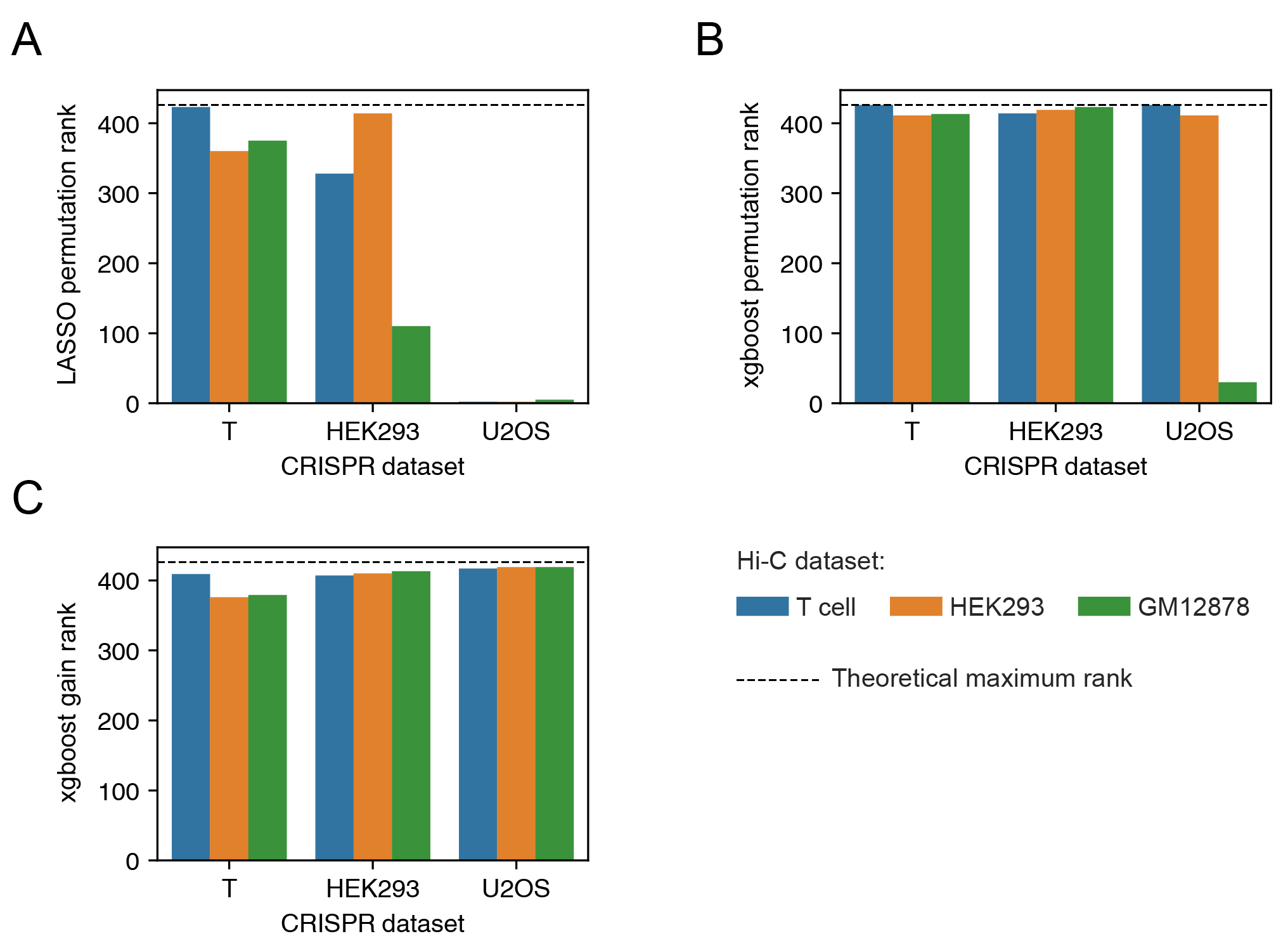

Supplement: S7 Fig — Average importance ranks of the 3D features compared to other features, over 1000 model iterations. Blue/orange/green bars represent 3D features generated from Hi-C in T/HEK293/GM12878 cells, respectively. The dashed line represents the theoretical rank of a feature ranked first in all 1000 models. (A) Permutation importance in LASSO models. (B) Permutation importance in xgboost models. (C) Gain importance in xgboost models. (TIF) [file pcbi.1012214.s014.tif]

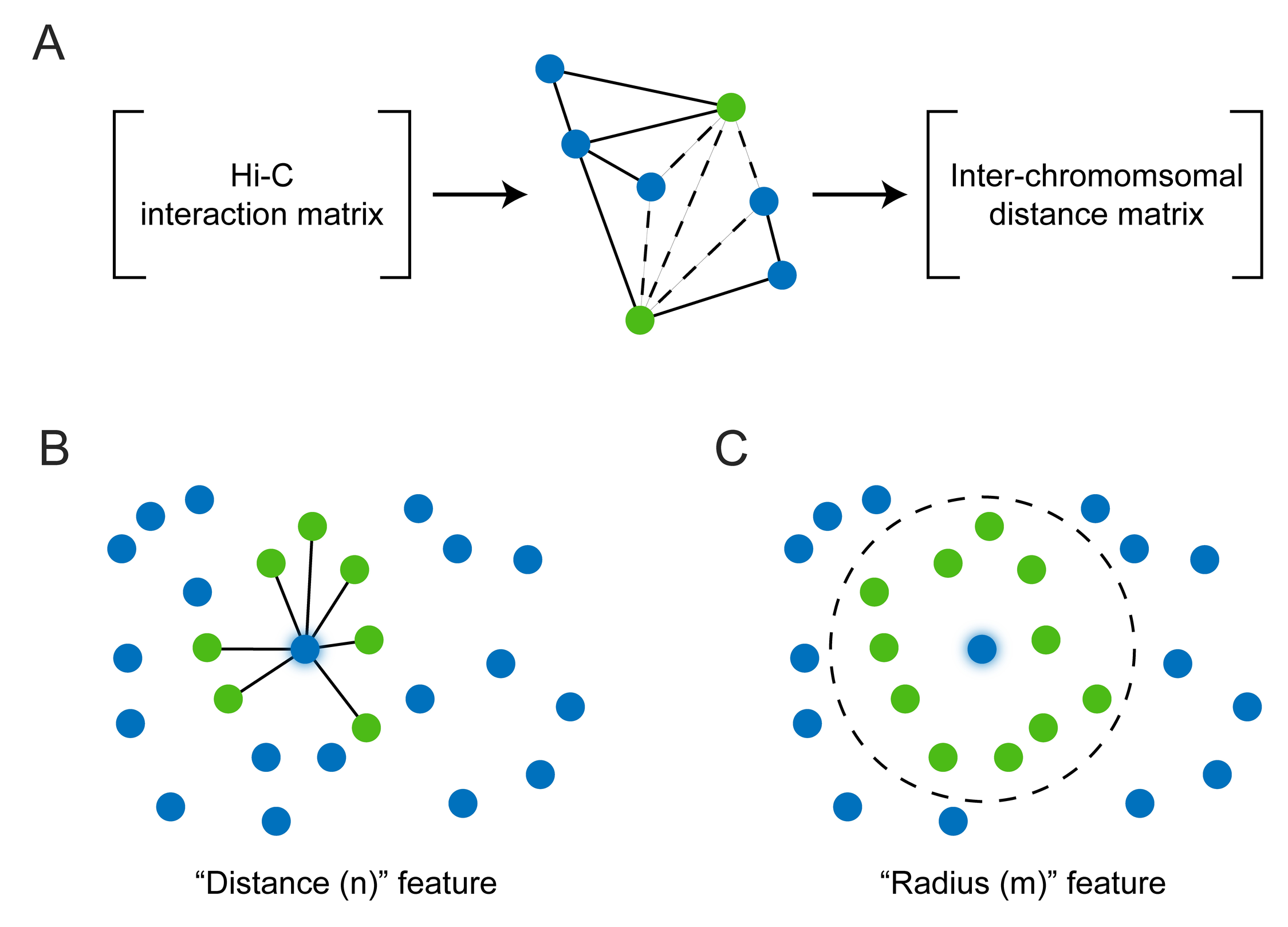

Supplement: S8 Fig — (A) The pipeline for estimating inter-chromosomal distances. We converted each chromosomal interaction matrix (left) to a weighted graph (center), where each node is a 10kb genomic region, and an edge represents an interaction between two regions. The weight of each edge is 1 / (number of interactions). Next, for each pair of regions on the same chromosome (green nodes), we found the p shortest paths between them (dashed edges) and took the distance between them to be the average weight of these paths (right). (B) An illustration of the “Distance (n)” feature–for a given genomic region (highlighted blue), we found the n closest regions (green), and calculated their average distance from it. (C) An illustration of the “Radius (m)” feature–for a given genomic region (highlighted blue), we counted the number of nodes (green) closer to it than a certain radius. The radius was determined relatively to all the inter-chromosomal distances found (e.g. the distance in the top 10%). (TIF) [file pcbi.1012214.s015.tif]
